# Real-world outcomes of ketogenic diet therapy in children with drug-resistant epilepsy in a prospective cohort study

**DOI:** 10.1038/s41598-026-42913-1

**Published:** 2026-03-05

**Authors:** Rui Han, Yiran Xu, Jun Sun, Qiliang Guo, Kaixian Du, Yan Dong, Xiaoli Li, Lingling Zhang, Hao Chen, Lin Li, Dan Xu, Jiajia Duan, Bingbing Li, Xiaoli Zhang, Changlian Zhu

**Affiliations:** 1 Pediatric Neurology, Department of Pediatrics, Third Affiliated Hospital of Zhengzhou University, Zhengzhou, 450052 Henan China; 2 Henan Key Laboratory of Child Brain Injury and Henan Pediatric Clinical Research Center, Institute of Neuroscience, Third Affiliated Hospital of Zhengzhou University, Zhengzhou, 450052 Henan China; 3 Department of Pediatrics, Third Affiliated Hospital of Zhengzhou University, Zhengzhou, 450052 Henan China; 4 Department of Neonatology, Third Affiliated Hospital of Zhengzhou University, Zhengzhou, 450052 Henan China; 5 Department of Women and Children’s Health, Karolinska Institute, Stockholm, 17176 Sweden; 6 Center for Brain Repair and Rehabilitation, Institute of Neuroscience and Physiology, University of Gothenburg, Gothenburg, 405 30 Sweden

**Keywords:** Ketogenic diet, Drug-resistant epilepsy, Seizure control, Electroencephalogram, Cognitive improvement, Diseases, Medical research, Neurology, Neuroscience

## Abstract

The ketogenic diet (KD) is an established non-pharmacological treatment for drug-resistant epilepsy (DRE) in children, but data from large prospective cohorts in China remain limited. We conducted a prospective observational study of children with DRE whose caregivers elected KD treatment compared with those who continued conventional therapy. Seizure frequency, EEG findings, cognitive assessments, and adverse events were monitored over six months. A total of 136 children were enrolled (73 KD; 63 controls). At six months, children receiving KD showed a higher proportion of seizure reduction ≥ 50% compared with controls. EEG evaluations revealed improvements in background activity and reduction in epileptiform discharges, and cognitive assessments demonstrated gains in specific domains. Adverse effects were generally mild and manageable with dietary adjustments. In this single-center prospective cohort, KD was associated with improved seizure control, EEG patterns, and cognitive performance over six months compared with conventional therapy. While limited by non-randomized design and short follow-up, these findings provide real-world evidence supporting KD as a feasible and safe adjunctive therapy for pediatric DRE in China. Longer randomized studies are warranted to establish causality and evaluate long-term outcomes.

## Introduction

Epilepsy is one of the most common neurological disorders in children and approximately one-third of affected patients do not achieve satisfactory seizure control despite treatment with anti-seizure medications drugs (ASMs)^1,2^. This condition, termed drug-resistant epilepsy (DRE), is defined by the International League Against Epilepsy (ILAE) as the failure trials of at least two well-tolerated and appropriately chosen ASMs regimens to achieve sustained seizure freedom^3^. DRE significantly impacts patients’ quality of life, often leading to cognitive impairments, developmental delays, and social difficulties, which impose substantial psychological and economic burdens on affected families^4^.

In recent years, the ketogenic diet (KD) has gained increasing recognition as a non-pharmacological treatment for DRE, particularly in children^5,6^. KD - a high-fat, low-carbohydrate dietary therapy - induces a metabolic shift towards ketone body production, providing an alternative energy source for the brain. Growing evidence suggests that KD not only reduces seizure frequency but also exerts neuroprotective effects, potentially improving cognitive function and behavioral outcomes^7–9^. Unlike traditional ASMs, KD alters brain metabolism by promoting the production of ketone bodies, which serve as an alternative energy source, thereby reducing neuronal hyperexcitability and modulating key pathways involved in seizure generation^8^. Adjusting the composition allows KD to balance between reducing seizures and relatively few adverse events^10,11^. Despite these promising findings, the mechanisms underlying its efficacy remain incompletely understood, and systematic studies evaluating its clinical impact are limited.

Although previous studies have demonstrated the short-term efficacy of KD in seizure control, longitudinal data comparing its effects with conventional ASMs regimens remain limited^12^. Additionally, comparisons between KD and traditional ASMs regimens in children with DRE remain sparse. These gaps highlight the need for robust clinical research to elucidate the therapeutic potential of KD and its role as an adjunct to conventional treatments.

This prospective cohort study aims to evaluate the efficacy and safety of KD in children with DRE by examining its impact on seizure control, EEG changes, and cognitive function over a six-month period, compared to routine ASMs adjustments. By providing a comprehensive analysis of these clinical outcomes, our findings may contribute to the optimization of KD and inform evidence-based treatment strategies for pediatric DRE.

## Methods

### Study design and grouping

This study included DRE children treated at the Pediatric Neurology Department of the Third Affiliated Hospital of Zhengzhou University from March 2022 to December 2023. The trial was registered at the Chinese Clinical Trial Register (www.chictr.org.cn, No: ChiCTR2200056805, Registration date:2022-02-17). Participants were divided into two groups based on the preferences of their parents or guardians: a KD group and a control group. The KD group received a ketogenic diet in addition to their existing ASMs regimen, while the control group underwent adjustments to their ASMs, including dose modifications, addition of new ASMs, or glucocorticoid therapy as needed. Children who have received KD treatment in the past, children with an abnormal screening of hereditary metabolic diseases, and children with serious organic diseases (such as heart, liver, and kidney), severe mental diseases, and blood system diseases will be excluded from this study. Prior to study initiation, participants underwent a baseline observation period of one month (or two weeks for neonates with seizure frequencies exceeding two per day) to assess their baseline seizure frequency.

This study was approved by the Institutional Ethics Committee of the Third Affiliated Hospital of Zhengzhou University (Approval No. 2021-017-01). Written informed consent was obtained from the parents or legal guardians of all participants prior to enrollment.

Sample size calculation was based on a preset 10% difference in epilepsy control rate (45% in the KD group vs. 35% in the control group). Using PASS 2021 software (two-sided α = 0.05, power = 0.80), the minimum required sample size was calculated to be 128 participants (68 in the KD group and 60 in the control group). Considering a 5% dropout rate, the total sample size was increased to 136 participants (73 in the KD group and 63 in the control group).

### Ketogenic diet Intervention

Children in the KD group underwent a comprehensive pre-treatment assessment to assess baseline metabolic and health status. This included measurements of blood pressure, body weight, routine blood and urine tests, liver and kidney function, electrolytes, echocardiography, and renal ultrasound. Following assessment, individualized KD plans were developed in collaboration with pediatric neurologists and dietitians. The diet was initiated with a 1:1 ketogenic ratio, which was gradually increased to 2:1, 3:1, 4:1, or higher as tolerated. Before initiating the diet, parents and caregivers received structured training on preparing ketogenic meals, monitoring urine ketones and blood glucose, and tracking seizure activity and recognizing and managing potential adverse effects. KD treatment was discontinued if severe adverse effects occurred or if the patient was unable to tolerate the diet.

### Control group management

Children in the control group received tailored adjustments to their ASMs regimens. Changes included dose adjustments, introduction of new ASMs, or administration of glucocorticoids based on the type and severity of epilepsy. All modifications were guided by clinical evaluations and established treatment protocols.

### Data collection and follow-up

Baseline demographic and clinical data were collected, including age of seizure onset, gender, etiology, prior ASMs use, and baseline seizure frequency. Follow-up assessments were conducted at regularly scheduled intervals over six months, either during outpatient visits or via telephone consultations. Seizure frequency was recorded in seizure logs maintained by caregivers. The absolute number of seizures recorded during this baseline observation period was used to determine each patient’s baseline seizure frequency, which was then compared between groups and served as the reference for calculating subsequent percentage reductions. EEG and cognitive function were evaluated at baseline and after six months of treatment.

### Outcome measures

Seizure Control: Seizure outcomes were classified using the Engel Epilepsy Outcome Scale^13^, with class I (Complete control: no seizures), class II (Effective control: seizure reduction ≥ 90%), and class III (Incomplete control: seizure reduction ≥ 50%) considered effective. Class IV (Ineffective control: seizure reduction < 50%) was categorized as non-responsive.

EEG Improvement: Baseline and six-month EEGs (≥ 8-hour recordings including awake and sleep phases) were analyzed by two independent neurologists. EEG improvements were classified as interictal improvement, seizure-related improvement, or both.

Cognitive Function: Cognitive assessments included the Griffiths Developmental Scale (for children aged 0–8 years) or the Wechsler Intelligence Scale for Children (WISC) for older participants. Improvements in functional domains (e.g., gross motor, language, eye–hand coordination, personal–social, and performance) were classified as effective if changes exceeded predefined thresholds. An improvement in at least one functional domain of more than 2.5% (Griffiths Scale) or 5 points (WISC score) were considered effective. Overall cognitive improvement was defined as fulfilling the above criterion in at least one of the assessed domains or scales.

Safety and Tolerability: Adverse events, changes in height and weight, and laboratory parameters were monitored at baseline and during follow-up visits at 6 months. Reasons for KD discontinuation were documented, including intolerance or significant adverse effects.

### Statistical analysis

All statistical analyses were performed using SPSS 26.0 and R4.2.2. Continuous variables were assessed for normality using the Kolmogorov-Smirnov test. Normally distributed variables were reported as mean ± standard deviation and compared using Student’s t-test.

Skewed data were reported as median (interquartile range, IQR) and analyzed using the Mann-Whitney U test. Categorical variables were expressed as percentages and analyzed using the Chi-square or Fisher’s exact test. After adjusting confounding factors such as age of onset, gender, and disease duration, logistic regression analysis was used to explore the impact of KD on seizure control, EEG improvement, and cognitive function. To control for potential confounders, multivariable logistic and Cox regression analyses were performed, adjusting for sex, age at seizure onset, disease duration, number of ASMs used prior enrollment, and etiologic category. Logistic regression was used to evaluate the independent effect of KD on seizure control, EEG improvement, and cognitive function, with results expressed as odds ratios (OR) with 95% confidence intervals (CI) and p-values.

Time-to-event analysis was performed using Kaplan–Meier curves and Cox proportional hazards regression. The event was defined as achieving effective seizure control (Engel class I–III), and children who had not achieved seizure control by the end of the 6-month follow-up were treated as right-censored observations. Kaplan–Meier curves were plotted to show the probability of not yet achieving effective seizure control. Group differences were assessed using Cox regression with the control group as the reference, and results were expressed as hazard ratios (HR) with 95% CI and p-values. A p-value < 0.05 was considered statistically significant.

## Results

### Baseline characteristics

**Fig. 1:**
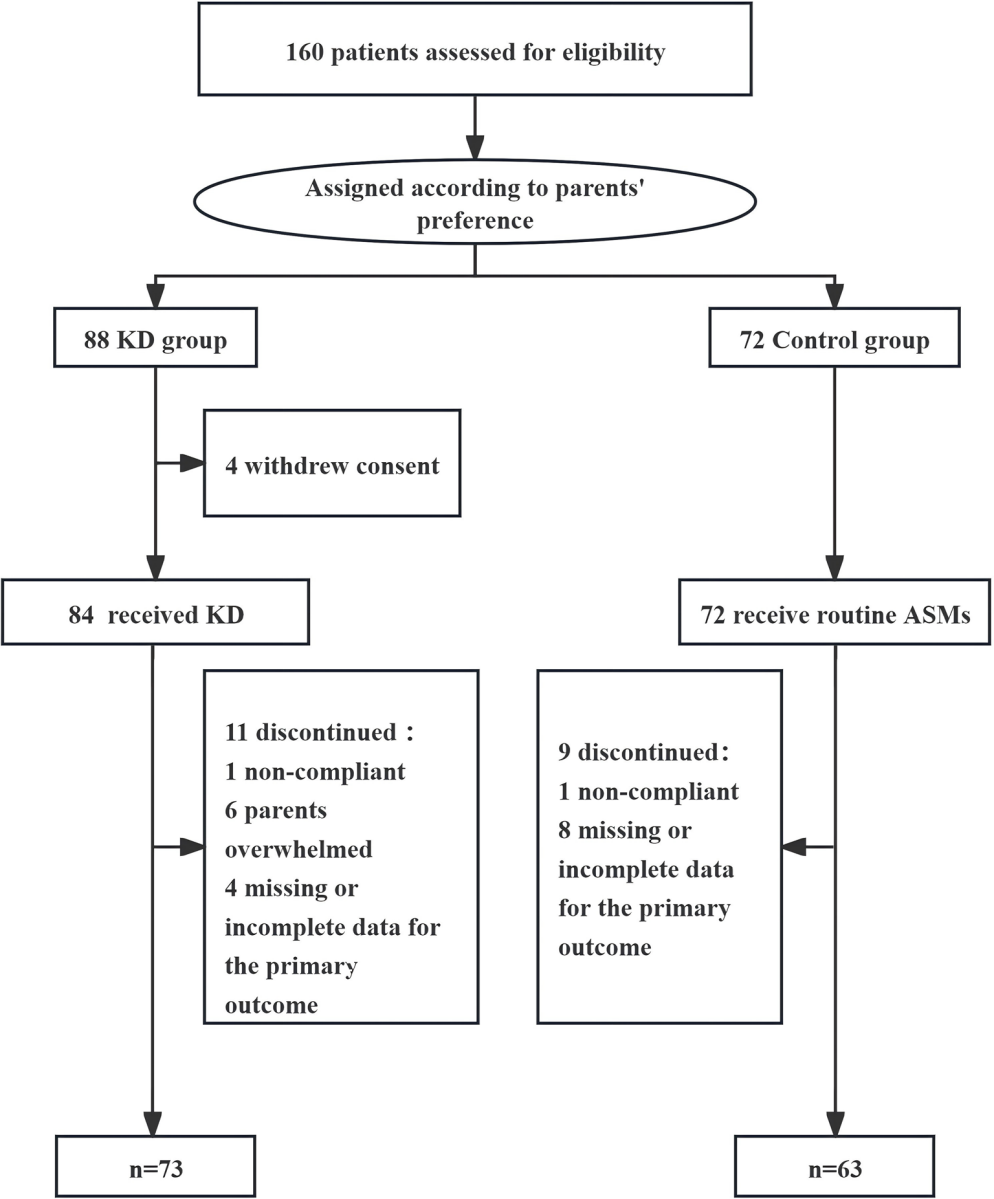
Flow diagram of participant enrollment and grouping. Flowchart showing participant screening, group allocation, and follow-up. Of 136 children with drug-resistant epilepsy, 73 entered the ketogenic diet (KD) group and 63 the control group. Grouping was based on caregiver preference. Follow-up was conducted over six months.

The study included 136 children, with 73 assigned to the KD group and 63 to the control group (Fig. 1). Baseline demographic characteristics (Table 1), including age of seizure onset, gender distribution, etiology, baseline seizure frequency, seizure type, number of seizure type, types of ASMs, disease duration, and the changes of weight and height were comparable between the two groups. Although the KD group had a higher proportion of hereditary/metabolic etiologies, these differences did not significantly affect the overall comparability of the groups.

**Table 1:** Baseline demographic and clinical characteristics of the KD and control groups.

	Control group (*n* = 63)	KD group (*n* = 73)	*P*
Age of onset (year)	0.92(0.42–2.25)	0.80(0.55–1.26)	0.84
Gender			0.99
Male (%)	60.3(38/63)	60.3(44/73)	
Female (%)	39.7(25/63)	39.7(29/73)	
Etiology			0.06
Hereditary/Metabolic (%)	23.8(15/63)	39.7(29/73)	
Structural (%)	47.6(30/63)	26.0(19/73)	
Infection/Immunity (%)	4.8(3/63)	4.1(3/73)	
Unknown (%)	23.8(15/63)	30.1(22/73)	
Baseline seizure frequency	90.00(13.00–96.00)	90.00(90.00–99.00)	0.43
Seizure type			0.14
Focal (%)	27.3(38/139)	20.3(32/158)	
Spasms (%)	25.9(36/139)	34.8(55/158)	
Tonic (%)	3.6(5/139)	8.9(14/158)	
Clonic (%)	2.2(3/139)	3.2(5/158)	
Myoclonic (%)	10.8(15/139)	8.9(14/158)	
Absence (%)	9.4(13/139)	3.8(6/158)	
Tonic-clonic (%)	10.1(14/139)	10.8(17/158)	
Tonic-spasms (%)	7.2(10/139)	4.4(7/158)	
Status epilepticus (%)	3.6(5/139)	5.1(8/158)	
No. Seizure type (IQR)	2.00(1.00–3.00)	2.00(1.00–3.00)	0.64
Disease duration (year)	2.50(0.75-4.00)	2.40(1.90–3.65)	0.18
Types of ASMs (species)	4.00(3.00–5.00)	4.00(3.00–5.00)	0.46
Change of Weight (Kg)	0.50(0.00-1.50)	1.00(0.50–1.83)	0.24
Change of Height (cm)	3.00(2.00–6.00)	4.00(2.00–7.00)	0.25

The total number of KD groups does not add up to 158, and the total number of control groups does not add up to 139, because some participants do not know that there are two or more seizure types.

### Seizure control

**Fig. 2:**
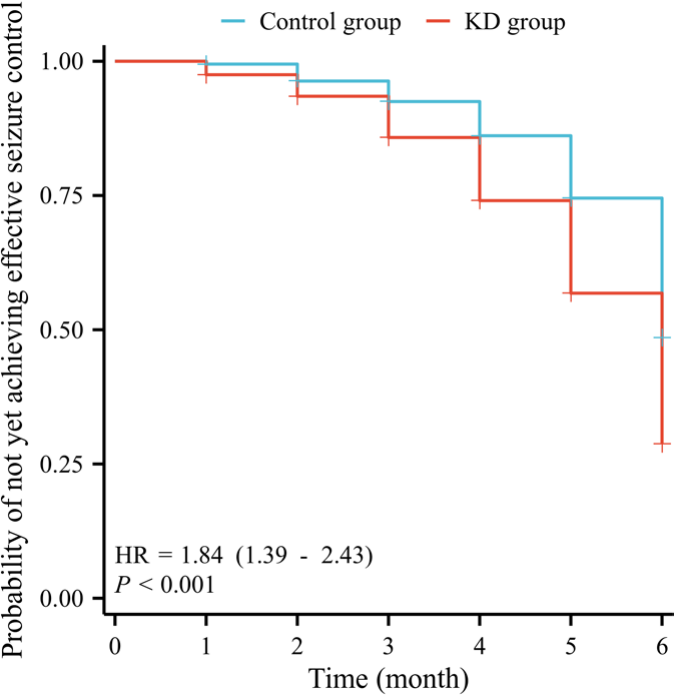
Kaplan–Meier curve for seizure control over time. Kaplan–Meier analysis comparing the probability of effective seizure control (Engel class I–III) between the KD and control groups over six months. The KD group showed significantly better outcomes (HR = 1.84, 95% CI 1.39–2.43, *p* < 0.001).

The KD group demonstrated superior seizure control outcomes compared to the control group. During the 6-month follow-up period, the overall effective seizure control rate (Engel class I–III) was significantly higher in the KD group (49.3% vs. 35.0%) (Table 2). Logistic regression analysis showed that, except for the second month, KD significantly increased the probability of effective seizure control at all other time points. Although the complete seizure control rate (Engel class I) in the control group was higher than that in the KD group during the second month, and it improved over time in both groups. Kaplan–Meier analysis showed that the KD group had a lower probability of remaining without effective seizure control over time compared with the control group (Fig. 2), indicating a higher proportion of patients who achieved seizure control. Cox proportional hazards regression further confirmed that KD treatment was associated with a higher likelihood of seizure control (HR = 1.84, 95% CI 1.39–2.43, *p* < 0.001). Minor differences between the survival probabilities shown in Fig. 2 and the raw percentages in Table 2 reflect right-censoring adjustment inherent to survival analysis.

**Table 2:** Seizure control effectiveness in both groups.

	Effective				Ineffective (IV)		*P*	OR (95%CI)
	Complete control (I)		Reduce by 50%-99% (II + III)					
	Control group	KD group	Control group	KD group	Control group	KD group		
1st month (%)	0.0(0/63)	5.5(4/73)	3.2(2/63)	9.6(7/73)	96.8(61/63)	84.9(62/73)	0.05	5.41(1.15,25.43)*
2nd month (%)	4.8(3/63)	4.1(3/73)	11.1(7/63)	16.4(12/73)	84.1(53/63)	79.5(58/73)	0.67	1.37(0.57,3.31)
3rd month (%)	6.3(4/63)	9.6(7/73)	9.5(6/63)	23.3(17/73)	84.1(53/63)	67.1(49/73)	0.06	2.60(1.13,5.98)*
4th month (%)	7.3(5/63)	12.3(9/73)	12.7(8/63)	28.8(21/73)	79.4(50/63)	58.9(43/73)	0.03	2.68(1.25,5.78)*
5th month (%)	12.7(8/63)	13.7(10/73)	14.3(9/63)	32.9(24/73)	73.0(46/63)	53.4(39/73)	0.03	2.36(1.15,4.86)*
6th month (%)	17.5(11/63)	15.1(11/73)	17.5(11/63)	34.2(25/73)	65.0(41/63)	50.7(37/73)	0.01	1.81(0.91,3.62)*

Note: *: After adjusting for confounding factors, logistic regression analysis showed a statistically significant difference (*P* < 0.05).

### EEG improvement

EEG analyses revealed significant improvements in the KD group compared to the control group (Table 3). At six months, the KD group exhibited a higher proportion of patients with interictal and seizure-related EEG improvements (31.5% vs. 6.3%). The KD group also had a significantly lower percentage of patients with no EEG improvement (42.5% vs. 69.8%). Logistic regression analysis showed that the probability of EEG improvements in KD group was 2.827 times that in control group (OR = 2.83, 95% CI: 1.32–6.06, *p* = 0.001). These findings highlight the positive impact of KD on normalizing brain electrical activity in children with DRE.

**Table 3:** EEG and cognitive function improvements in the KD and Control groups over 6 months.

	Control group (*n* = 63)	KD group (*n* = 73)	OR (95%CI)	*P*
EEG			2.83 (1.32–6.06)	<0.001
Seizure improvement	7.9 (5/63)	11.0 (8/73)		
Interictal improvement	15.9 (10/63)	15.1 (11/73)		
Interictal and seizure improvement	6.3 (4/64)	31.5 (23/73)		
No improvement	69.8 (44/63)	42.5 (31/73)		
**Cognitive result**				
Overall cognitive improvement	19.0 (12/63)	35.6 (26/73)	2.79 (1.17–6.43)	0.03
Griffths developmental domains (0–8 years)				
Gross motor improvement	10.9 (5/46)	28.8 (15/52)	3.32 (1.10–10.04)	0.04
Personal-social improvement	8.7 (4/46)	25.0 (13/52)	3.50 (1.05–11.65)	0.09
Language improvement	13.0 (6/46)	30.8 (16/52)	2.96 (1.05–8.39)	0.04
Eye-hand coordination improvement	8.7 (4/46)	25.0 (13/52)	3.50 (1.05–11.65)	0.09
Performance improvement	10.9 (5/46)	28.8 (15/52)	3.32 (1.10–10.04)	0.04
WISC cognitive outcome (≥ 8 years)				
Full-Scale IQ increase ≥ 5 points	17.6 (3/17)	33.3 (7/21)	2.33 (0.50–10.91)	0.28

### Cognitive function

The KD group showed significantly greater improvements in cognitive outcomes compared to the control group (Table 3). After six months, the proportion of patients with measurable cognitive improvements was higher in the KD group (35.6% vs. 19.0%). Logistic regression analysis showed that the probability of overall cognitive improvement in the KD group was 2.79 times higher than in control group (OR = 2.79, 95% CI: 1.17–6.43, *p* = 0.031). Among children assessed with the Griffiths Scale, higher rates of improvement were observed across several domains—particularly gross motor, language, and eye–hand coordination (all *p* < 0.05). In older children assessed by WISC, an upward trend was also noted in Full-Scale IQ scores (33.3% vs. 17.6%), although this difference was not statistically significant. Overall, KD therapy was associated with broader cognitive gains beyond seizure control, indicating improvements across multiple functional domains.

### Safety and tolerability

KD treatment was generally well-tolerated, with most adverse events being mild and manageable. No severe adverse events were reported, and treatment adherence remained high throughout the study. A total of 22 mild or moderate adverse events were reported in control group and 29 were reported in KD group (Table 4). The distribution of adverse events across different system organ classes, as categorized by the Medical Dictionary for Regulatory Activities (MedDRA), showed no significant differences between the two groups. Reasons for discontinuing KD included difficulty maintaining the diet and caregiver-reported challenges in meal preparation.

**Table 4:** Adverse events in the KD and control groups over 6 months.

	Control group (*n* = 63)	KD group (*n* = 73)	*P*
Number of adverse events	22	29	
MedDRA system organ class:			0.26
Gastrointestinal disorders	2/22	5/29	
Diarrhea	0	2	
Nausea	2	1	
Vomiting	0	2	
Investigations	6/22	8/29	
Fibrinogen decreased	6	6	
Cholesterol high	0	2	
Metabolism and nutrition disorders	7/22	13/29	
Anorexia	3	2	
Hypertriglyceridemia	0	10	
Hypokalemia	1	1	
Hyponatremia	3	0	
Nervous system disorders	5/22	1/29	
Dizziness	1	0	
Headache	1	0	
Somnolence	3	1	
Psychiatric disorders	2/22	1/29	
Agitation	1	0	
Restlessness	1	1	
Renal and urinary disorders	0/22	1/29	
Renal calculi	0	1	

## Discussion

The findings of this study underscore the significant therapeutic potential of KD as an adjunctive treatment for DRE in children. KD demonstrated superior efficacy in seizure control, EEG improvement, and cognitive function enhancement compared to conventional ASMs adjustments, with particularly notable benefits observed during the early stages of treatment.

Existing literature supports KD as an effective intervention for DRE. A previous meta-analysis indicated that the KD was more effective than conventional treatments in the short term (≤ 3 months), reducing seizures by 50% or more^14^. In line with previous studies, our findings show that KD group achieved a significantly higher overall effective rate of seizure control (49.3% vs. 35.0%). The KD group particularly achieve early and substantial seizure control, with 15.1%(class I + II +III)in the first months compared to 3.2% in the control group. These rapid effects may be explained by KD-induced metabolic adaptations, such as increased availability of ketone body, modulation of neuronal excitability, and improved mitochondrial efficiency, which collectively raise seizure thresholds^15–21^.

Beyond idiopathic and structural epilepsies, several genetic and metabolic forms of epilepsy—including glucose transporter type 1 deficiency syndrome and pyruvate dehydrogenase complex deficiency—have been shown to respond favorably to KD therapy^22,23^. These disorders share impaired cerebral energy metabolism as a key pathophysiological mechanism, making KD a rational therapeutic approach by providing ketone bodies as an alternative energy substrate^24,25^. Although only a small proportion of hereditary or metabolic etiologies were represented in our cohort, future genotype-specific analyses may further clarify the underlying therapeutic mechanisms and help optimize KD as a precision treatment strategy for pediatric epilepsy.

The improvements observed in EEG parameters further highlight KD’s role in modulating brain activity^26,27^. Previous studies have reported marked reductions in epileptiform discharges following KD treatment^28,29^. In our cohort, a higher proportion of patients in the KD group exhibited interictal and seizure-related EEG improvements (31.5% vs. 6.3%), findings that are consistent with KD’s proposed neuroprotective and network-stabilizing effects. These changes may serve as potential biomarkers for KD’s efficacy and help clinicians monitor treatment response^30,31^.

In addition to seizure reduction and EEG normalization, KD was also associated with improved cognitive outcomes^32,33^. Approximately 35.6% of children in the KD group showed measurable cognitive gains, consistent with prior evidence suggesting that KD may enhance brain function through improved energy metabolism and synaptic plasticity^34^. However, these improvements should be interpreted with caution, as they may reflect indirect effects mediated by reduced seizure frequency and improved interictal EEG activity, rather than direct neurocognitive actions of KD ^32,35^. A reduced seizure burden and normalization of brain electrical activity are both known to enhance attention, memory, and overall neurodevelopmental outcomes in children with epilepsy. Future studies employing mediation analyses and neurophysiological biomarkers will be required to delineate direct versus seizure-related pathways underlying cognitive improvement during KD therapy.

While KD showed superior efficacy, its benefits diminished slightly over time relative to the control group, which exhibited improved seizure outcomes in later months—possibly due to ASMs optimization. Nevertheless, the KD group maintained higher overall seizure remission rates throughout the study, highlighting its sustained therapeutic effects when applied consistently. Previous evidence indicates that longer KD treatment durations may promote more stable metabolic adaptation and seizure remission^36^. Li et al. found that in assessing the long-term efficacy of KD treatment for children with DRE, a maintenance period of less than 12 months was significantly associated with an increased risk of relapse^37^. This suggests that in cases of shorter treatment durations, patients may not have had enough time to fully establish stable ketone body metabolism, which could lead to a relapse of seizures.

Despite its promising results, this study has several important limitations. The single-center, non-randomized design—in which treatment allocation was determined by caregiver preference—may have introduced potential selection bias. Baseline etiologic imbalance (a higher proportion of hereditary/metabolic cases in KD group and more structural causes in control group) could have influenced treatment response, although major confounders were accounted for in the multivariable models. Absolute baseline seizure frequencies have been added to provide better context for interpreting seizure reduction outcomes. Furthermore, the relatively short 6-month follow-up period limits the ability to evaluate long-term efficacy and tolerability. Future large, multicenter studies with longer observation and mechanistic endpoints are warranted to confirm these findings and to further elucidate the clinical and neurobiological effects of KD.

## Conclusions

Our findings suggest that KD may offer meaningful benefits in seizure reduction, EEG activity, and cognition for children with DRE in a real-world clinical setting. Importantly, adverse effects were generally mild and manageable, indicating that KD is feasible in routine practice when supported by trained caregivers and multidisciplinary teams. However, given the observational design and six-month follow-up, the results should be interpreted with caution. Future randomized controlled trials and longer follow-up are needed to validate these associations and to better define the long-term efficacy and safety of KD in diverse pediatric populations.

## Data Availability

The datasets generated and/or analysed during the current study are available from the corresponding author for non-commercial research purposes, in anonymized form, and in accordance with ethical approval and data protection regulations. There are no restrictions on access other than those required to protect participant confidentiality.
